# Supplementary figures and images for: Characterization and Future Distribution Prospects of “Carciofo di Malegno” Landrace for Its In Situ Conservation
Source: Plants (Basel). 2024 Feb 28;13(5):680. doi: 10.3390/plants13050680 (PMC10935239; doi:10.3390/plants13050680)

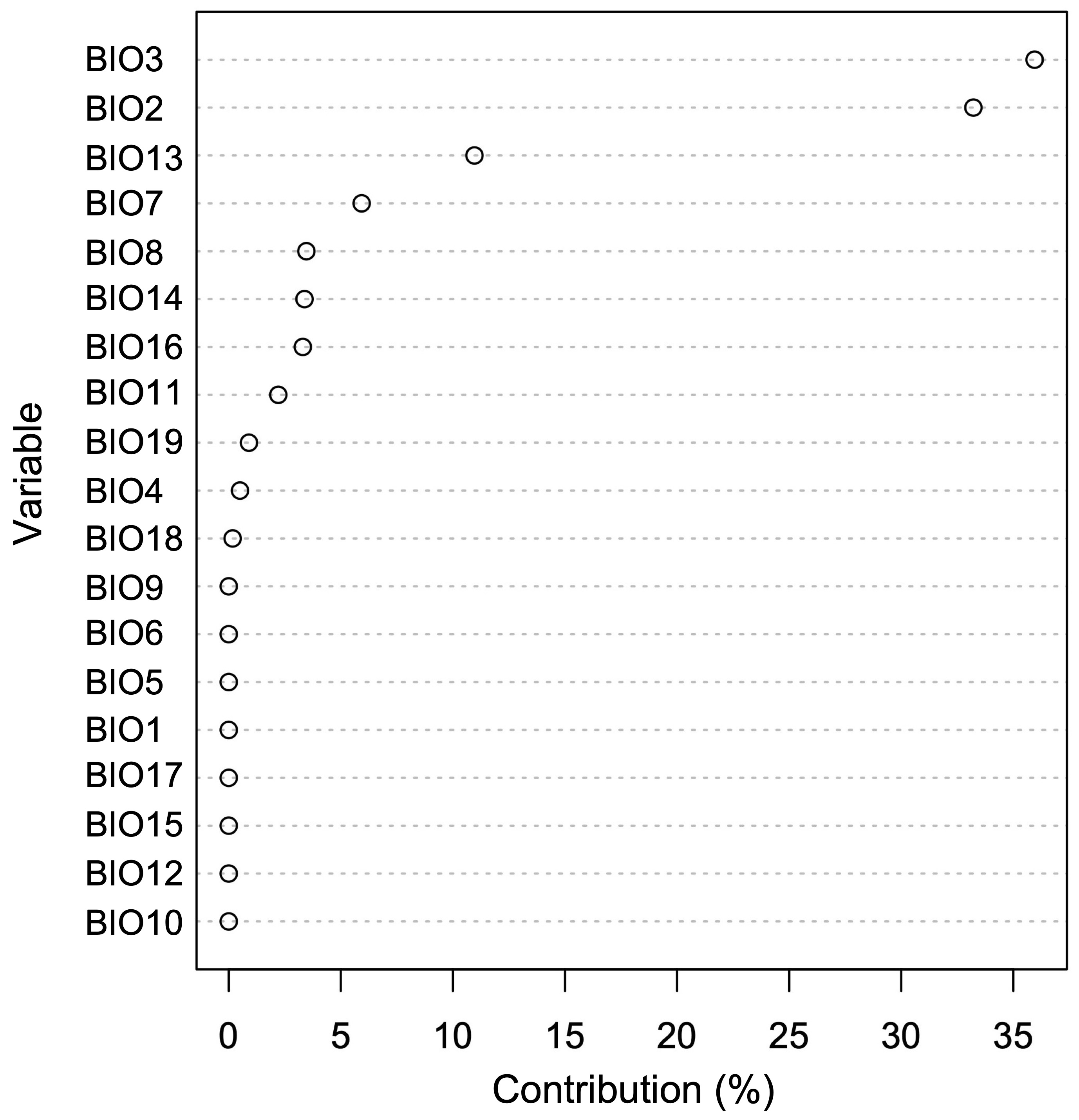

Supplement: Supplementary file 1 [file plants-13-00680-s001.zip › plants-2857214-supplementary.jpg]
